# Sciatica Relieved by Galvanism

**Published:** 1880-01

**Authors:** H. L. Wilson

**Affiliations:** Atlanta, Ga.


ATLANTA
Medical and Surgical Journal.

Vol. XVII.] JANUARY—1880. [No. 10.

### Original Communications.

### SCIATICA RELIEVED BY GALVANISM.

By H. L. WILSON, M.D., Atlanta, Ga.

Miss R , an intelligent, active and energetic young lady, just blooming into womanhood, had a severe attack of measles, from which she slowly recovered, never reaching her former health and vivacity, however. She was left in a nervous condition that kept her in doors, at first feeling languid and dull; at length she began to suffer more or less pain down the spine and lower extremities. Finally, a physician was called who, after hearing the history of her case, treated her back with counter-irritants, administering at the same time, nervous tonics. The treatment was only productive of partial relief and comfort; in fact, in a few months she began to grow worse in some respects. Her limbs felt at times, as though they were of immense weight—she could scarcely lift them—and the pain increased. It now became necessary to give opiates to get sleep by night, or quiet by day. Her's was now a pitiable condition; young, smart, pious and attractive, and among other interesting features, 'tis said her hand was sought by a young man in a neighboring State, whose bright and prosperous future, was predicted by all who knew him. Yet for eighteen long months, this pale faced, conscientious maiden held her faithful suitor in abeyance, refusing to be an invalid wife, even to the man she loved. Here was, truly, a field for the application of science, which alone could dispel the gloom and bid pain and suffering depart forever.

She reached Atlanta in October, 1879, and was taken to her room completely prostrated from a long ride upon the cars. She took morphia, ¼ gr., and retired. The next morning she was unable to get out of bed, and I repeated the morphia. Her appetite was gone, mucles flabby and weak; did not think of going out of the house, being in too much pain to move.

After hearing a full history of the case, I proceeded to make a thorough examination, and found the entire spine irritable and sensitive, especially on reaching the sacro iliac region, where I found pain on pressure; this acute tenderness extending to the right knee, the left being not so bad—nothing, save opiates, seemed to give relief. Believing that the back trouble was the result of nervous congestion, I determined to try a twenty-four (24) cup galvanic battery, made by the Galvano-Faradic M. F. C. I applied one sponge at the nape of the neck and the other on the sacrum. I used this daily, together with tonics for general-depression. She began to improve in one week; in one month she went anywhere she desired, walking with but little inconvenience. Her appetite returned, her muscles grew and she slept well without opiates.

At the end of nine weeks, she returned to her home apparently relieved entirely of all symptoms of sciatica The treatment is so simple and easy, that I am certain it will repay any one who will try it, and I am confident the result will be gratifying.

